# Ascorbic acid does not necessarily interfere with the electrochemical detection of dopamine

**DOI:** 10.1038/s41598-022-24580-0

**Published:** 2022-11-23

**Authors:** Samuel Rantataro, Laura Ferrer Pascual, Tomi Laurila

**Affiliations:** 1 Department of Electrical Engineering and Automation, Aalto University, 02150 Espoo, Finland; 2 Department of Chemistry and Materials Science, Aalto University, 02150 Espoo, Finland

**Keywords:** Biosensors, Bioanalytical chemistry

## Abstract

It is widely stated that ascorbic acid (AA) interferes with the electrochemical detection of neurotransmitters, especially dopamine, because of their overlapping oxidation potentials on typical electrode materials. As the concentration of AA is several orders of magnitude higher than the concentration of neurotransmitters, detection of neurotransmitters is difficult in the presence of AA and requires either highly stable AA concentration or highly selective neurotransmitter sensors. In contrast to the common opinion, we show that AA does not always interfere electrochemical detection of neurotransmitters. The decay of AA is rapid in cell culture medium, having a half-time of 2.1 hours, according to which the concentration decreases by 93% in 8 hours and by 99.75% in 18 hours. Thus, AA is eventually no longer detected by electrodes and the concentration of neurotransmitters can be effectively monitored. To validate this claim, we used unmodified single-wall carbon nanotube electrode to measure dopamine at physiologically relevant concentration range (25–1000 nM) from human midbrain organoid medium with highly linear response. Finally, AA is known to affect dopamine oxidation current through regeneration of dopamine, which complicates precise detection of small amounts of dopamine. By designing experiments as described here, this complication can be completely eliminated.

## Introduction

Electrochemical measurement system records currents arising from redox reactions occurring at the interface between electrode and electrolyte. This system can be used to measure concentration of analytes, such as neurotransmitters or AA, because electrochemical currents are directly proportional to concentration of the analyte that is being oxidized^1^. If more than one molecule undergoes electrochemical reactions at similar potential, currents from these two reactions are summed together. Electrochemical currents arising from the other molecules interfere detection of the analyte-of-interest. This is especially problematic when the concentration of interfering molecule is significantly larger than the analyte-of-interest.

Neurotransmitters are biomolecules that enable signal transduction between neurons and neuronal networks. The frequency of neurotransmitter release events depends on neuronal subtype and can be up to 20 Hz for dopaminergic neurons^2,3^, thus requiring sensors with high temporal resolution. Because electrochemical sensors can deliver both high sensitivity and temporal resolution, they are most prominent candidate for detecting neurotransmitters both in vivo and in vitro.

Carbon based materials are typically selected for electrochemical electrodes when neurotransmitters are desired to be measured, most significantly for the detection of dopamine. However, the presence of AA interferes with neurotransmitter detection due to its oxidation potential overlapping with the analyte-of-interest. Although dopamine concentration in the vicinity of synaptic cleft may be up to 1 μM^4^, we must note that the volume of synaptic cleft is exceedingly small ($\approx$ 0.0018 μm^3^ calculated by us based on synaptic cleft width being 25 nm^5^ and synaptic apposition surface area being 0.058–0.089 μm^2^^6,7^), which causes dopamine concentration to dilute rapidly as a function of distance from synaptic cleft. Thus, AA concentration can be 200–10,000 fold higher than dopamine concentration at the electrode surface, assuming the AA concentration being 200 μM in vitro^8^ and 100–250 μM in vivo^9^. Thus, even minor changes in AA concentration would make quantification of neurotransmitter levels impossible if the electrode surface is not thoroughly modified to be selective.

Common surface modification strategies include the use of ion-separation layers, such as Nafion, that prevent the diffusion of negatively charged ions (including AA) to the electrode surface, or alternatively application of specifically selected conductive materials (polymers or nanomaterials) that inherently separate the oxidation potential of dopamine and AA. These surface modifications typically decrease temporal resolution due to impairing diffusion of neurotransmitters from electrolyte to electrode surface^10,11^, whereas sensitivity towards dopamine may also decrease because of removing the highly-sensitive carbonaceous surface by presenting different material layer to the electrode-liquid interphase. In theory, surfaces may also be functionalized to improve selectivity between analytes without impairing temporal resolution, however the nature of relationships between functionalization and electrochemical response are largely unknown. Thus, modifying surfaces chemically to obtain a specific desired interaction is far from trivial.

It is widely known that AA interferes with the detection of dopamine through two mechanisms^12^. Dopamine and AA oxidize at similar potentials on most electrode materials, causing the recorded dopamine oxidation signal being directly interfered by the presence of AA. Secondly, AA is a powerful antioxidant and it can reduce the oxidation product of dopamine, dopamine-o-quinone, back to dopamine. This regeneration of dopamine increases its total available concentration at the electrode surface, causing dopamine oxidation current also being indirectly dependent on AA concentration^12^.

Because of the widely accepted truth about AA interference in vivo, it is often mistakenly also assumed that AA interferes all in vitro measurements. However, the conditions for AA metabolism differ significantly in vivo and in vitro, despite the fact that AA functions as an antioxidant and is thus sacrificially oxidized in both systems. In vivo, the oxidized form of AA can be enzymatically reduced back to L-ascorbic acid by dehydroascorbate reductase (DHAR)^13^. In addition to that, the presence of stronger antioxidants such as glutathione in large concentration in vivo can sacrificially oxidize instead of AA. By the combination of these two factors, concentration of AA remains high in vivo. The situation is different in vitro, where the concentration of stronger antioxidants in complete supplemented culturing media is commonly less than 1 μM (1:100 dilution^8^ of serum-free B-27 supplement^14^), but also because the functioning of DHARs is at least partly impaired due to the enzymatic reaction being dependent on glutathione^13^. Lastly, plasma contains chelators that decrease concentration of free metal ions and thus inhibits autoxidation of AA^15^.

Oxidation of AA in culture medium is thought to occur through autoxidation^16^ mediated by hydroxyl radicals^17^ that are produced from superoxide anions with a reaction catalyzed by iron ions^15^. However, we cannot rule out the contribution of oxidation arising from the antioxidative nature of AA against free radicals or its function as a reducing agent to various biomolecules^16^. Upon acting as an antioxidant or reducing agent, AA becomes oxidized in the process and thus cannot undergo electrochemical oxidation reaction at the electrode surface. All the aforementioned factors together cause the concentration of AA to decrease rapidly in culture media as a function of time^18^, with or without the presence of cells. Although this decay of AA in culture medium has been observed earlier by microfluorometry^18^, our present study replicates these results for the first time with electrochemical techniques but also more importantly, acknowledges this decay from the viewpoint of the interfering nature of AA.

Various surface-modified carbon electrodes have been shown to be selective against ascorbic acid, however these results are obtained with experimental designs that do not depict the real situation in vivo or in vitro. Most commonly, dopamine is used in the concentration of hundreds of μM^19–21^, a concentration that is 100–1000 fold higher than that observed by the release of cells. Alternatively, exceedingly slow scan rate^22^ or inherently slow electrochemical techniques such as differential pulsed voltammetry^23^ can be selected, which naturally increase selectivity between analytes but simultaneously also decrease temporal resolution to values where detection of transient dopamine release events is impossible.

Fast-scan cyclic voltammetry (FSCV) is a highly sensitive electrochemical technique where the recorded signal is background-subtracted, causing the elimination of stable interfering currents^24^. However, background-subtraction is simultaneously also the largest disadvantage of FSCV because any drifting in the background current will induce distortion to the recording signal. Drifting of the background current is caused by any changes that occur in the electrochemical system, including the electrolyte itself^25^. This significantly complicates those in vitro FSCV recordings where AA is an essential component of the culture medium, because the background current is constantly changing as the AA concentration decreases as a function of time.

Lastly, selectivity is commonly studied based on the separation between redox peaks of different analytes. However, onset to redox reaction can begin at much smaller potential than the *peak* potential, especially if the kinetics are slow. Although redox currents at these onset potentials are much smaller than currents at the peak potential, large difference in the concentration of analytes can make these “onset currents” significant and thus overshadow currents from the other analyte(s). Despite observing large separation in peak oxidation potentials between dopamine and AA, by performing experiments with high dopamine concentration, the detection of dopamine in real situation may prove to be impossible because of the interfering onset currents from AA. Furthermore, simply separating dopamine and AA oxidation peaks from one another does not eliminate the interference arising from dopamine regeneration. By acknowledging all the aforementioned problems, it would be optimal if one could ignore the effect of interference from AA by experimental design if possible.

We observed that the concentration of AA decreased rapidly in culture medium. By acknowledging this decay profile of AA, we measured dopamine in the physiological concentration range (25–1000 nM) from human midbrain organoid medium that was incubated for 20.5 hours but initially contained 200 μM AA. Our results demonstrate that AA does not necessarily interfere dopamine detection in vitro, although the duration after which AA interference disappears may be slightly affected by the culturing medium composition and electrode type. We must emphasize that AA remains to be an interfering agent in situations where culture media are not used and one uses a simple salt solution such as artificial cerebrospinal fluid (aCSF) or phosphate buffer solutions instead. However, AA is rarely added to aCSF and thus it is not interfering neurotransmitter detection from ex vivo samples either.

## Results and discussion

### Stability of ascorbic acid

We selected two different types of electrodes to study the stability of AA, which were tetrahedral amorphous carbon (ta-C) electrode and SWCNT network electrode. As we have thoroughly characterized both ta-C^26,27^ and SWCNT electrodes^28^ earlier, we did not repeat those characterizations in this study. If the reader is interested on the characteristics of these electrodes, we kindly guide the reader towards those publications.

**Figure 1:**
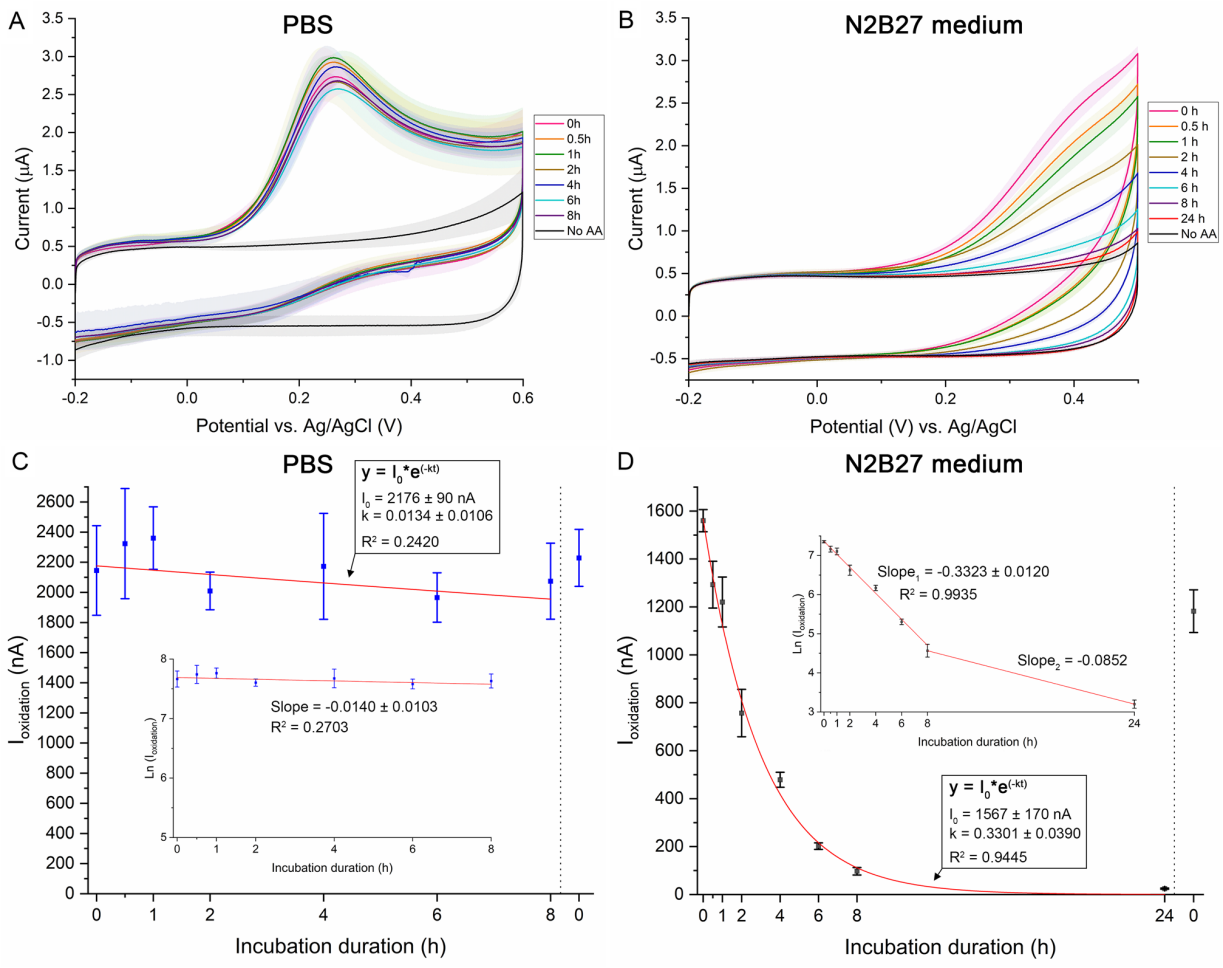
The concentration of ascorbic acid remains stable in PBS but decreases rapidly in culture medium, as is illustrated with cyclic voltammograms (**A**, **B**) and analyzed oxidation currents (**C**, **D**) at different incubation durations. Thick traces in (**A**, **B**) depict averaged trace (n = 3), whereas shaded region depicts SD. Analyzis graphs (**C**, **D**) show mean average AA oxidation current that is plotted with SD depicted by error bars. In N2B27 medium, the relationship between AA concentration and incubation duration followed accurately the pseudo first-order chemical reaction rate equation ($\hbox {R}^{2}$ = 0.9445). Linearization of the current (**D**, inlet) showed that this fit was near-perfect between 0 and 8 hour incubation duration, after which the fit broke down. In contrast to N2B27 medium, the goodness of fit was poor for AA oxidation in PBS ($\hbox {R}^{2}$ = 0.2420) and the variation in oxidation current at different time points was nearly within the SD of current at t = 0. The electrode type was SWCNT.

We observed with both electrode types that AA concentration did not significantly decay in PBS at pH 7.4 (Fig. 1 and Supplementary Fig. S1). The decrease in concentration was only 3.2 ± 2.8% after 8 hours as measured with SWCNT electrode and 4.0 ± 1.5% with ta-C, indicating that AA oxidation occurs very slowly when the oxidative species is oxygen from air. These results are in agreement with earlier results^29^, where AA autoxidation was shown to be inhibited in the abscence of catalytic metals.

As we performed experiments in N2B27 culture medium, which is the main component in human brain organoid culturing media^8^, we observed that the medium contains molecules that are oxidized at potentials smaller or similar to AA oxidation potential when ta-C was used (Supplementary Fig. S2). Thus, analyzing AA concentration in culture medium with ta-C electrodes was unreliable and because of that, all analysis from culture medium experiments were done with the SWCNT electrodes only.

In contrast to the situation in PBS, the concentration of AA in N2B27 medium decreased rapidly as a function of time (Fig. 1): AA concentration decreased by 87.0 ± 1.3% in 6 hours, by 93.7 ± 1.2% in 8 hours and by 98.6 ± 0.2 % in 24 hours, as measured. Because AA did not oxidize in PBS with the presence of oxygen but significantly decayed in culture medium, we conclude that majority of AA oxidation in culture medium occurs through the combination of metal-catalyzed autoxidation^16^, AA’s role as an antioxidant against oxidizing radicals^16^, and AA’s function as a reducing agent in biochemical reactions^30^.

In the N2B27 medium, concentration of free metal ions (0.935 μM of Fe & 0.003 μM of Cu) that catalyze AA autoxidation is not sufficiently large^31,32^ to be alone responsible for the observed rapid decay of AA. Thus, AA oxidation in culture medium must be caused mostly by both the presence of reactive oxygen species but also biomolecules that undergo oxidation due to the presence of oxygen. As the medium was under constant supply of oxygen from air into the medium, we may assume that the combined concentration of oxygen radical species and biomolecules that undergo oxidation is much larger than the concentration of AA. Thus, their concentration remains almost unchanged and the reaction rate is only dependent on AA concentration, enabling the use of pseudo first-order reaction equation to model the decay of AA in culture medium. The relation between measured oxidation current and pseudo first-order reaction rate is derived in Supplementary Information.

Accordingly, we fitted our oxidation current/incubation duration data with pseudo first-order chemical reaction equation and obtained good fit for the range between 0 and 24 hours ($\hbox {R}^{2}$ = 0.9445; Fig. 1D). To test that the reaction had pseudo first-order nature, we linearized the current/incubation duration relationship by applying natural logarithm to oxidation current data (Fig. 1D inlet). We observed fully linear relationship in the time range 0–8 hours ($\hbox {R}^{2}$ = 0.9935), after which the linearity broke down. While we cannot provide conclusive answer why this breakdown from linearity happened, we assume the oxidation reaction to also be dependent on other factors than simply AA once its concentration decreases notably, such as mass transfer. It could also be an artifact due to difficulties in obtaining oxidation current value when the cyclic voltammogram does not contain a clear oxidation peak and the curve appears highly similar to the medium without any analyte. Accordingly, we also tested fitting the current/incubation duration relationship data with pseudo first-order chemical reaction equation while leaving out the time point t = 24, obtaining a nearly perfect fit ($\hbox {R}^{2}$ = 0.9943, Supplementary Fig. S3).

Culture medium contains various molecules and proteins that may adsorb onto the electrode’s electrochemically active surface sites and block adsorption of AA to them, thus “fouling” the electrode and decreasing electrochemical currents. Because of this, we also measured oxidation current from freshly added AA after the measurement series to study, whether the decrease in oxidation current arises from surface fouling or from medium-induced AA oxidation. This is illustrated by the second “0h” time point in Fig. 1C and D. We detected oxidation current that was 75.8 ± 3.9% of that measured with fresh electrodes, indicating only minor fouling of the surface. Thus, we conclude that the near-complete disappearance of AA is not an artifact arising from electrode fouling.

Half-life stands for the time point where AA concentration has halved. By considering the direct proportionality between concentration and oxidation current as was noted in the Supplementary Information Eqs. S4–S6, we may calculate the half-life $\hbox {t}_{1/2}$ for AA as follows:

1 $$\begin{aligned} \frac{I_t}{I_0} = e^{-k_{exp}t_{1/2}} = 0.5 \quad \Longrightarrow \quad -k_{exp}t_{1/2} = \text{ln}(0.5) \quad \Longrightarrow \quad t_{1/2} = \frac{\text{ln}(0.5)}{-k_{exp}} \end{aligned}$$

where $\hbox {I}_{\mathrm{t}}$ denotes oxidation current at time point t and $\hbox {I}_{0}$ the current at time point t = 0, whereas $\hbox {k}_{\mathrm{exp}}$ denotes the experimentally determined rate constant.

Because of the deviation from linearity after 8 hours in culture medium, we calculated the half-life of AA based on the linearized slope between 0-8 hours where reaction rate constant $\hbox {k}_{\mathrm{exp}}$ = 0.3323. This yields half-life in culture medium to be 2.1 hours, replicating very closely to the half-life of $\approx$ 2 hours obtained elsewhere with microfluorometry^18^. However, we must note here that if the ratio between Petri dish surface area and medium volume ($\hbox {A}_{\mathrm{Surface}}/\hbox {V}_{\mathrm{medium}}$) decreases, we expect the half-life to slightly increase because of inhibited gas diffusion to the medium.

In contrast to the situation in culture medium, concentration of AA in PBS reaction did not follow pseudo first-order reaction ($\hbox {R}^{2}$ = 0.2420) and instead, remained stable throughout time period of 8 hours (Fig. 1A and C). This was not surprising because we used cell-culturing grade PBS with very low concentration of metallic impurities.

### Dopamine detection from organoid medium

**Figure 2:**
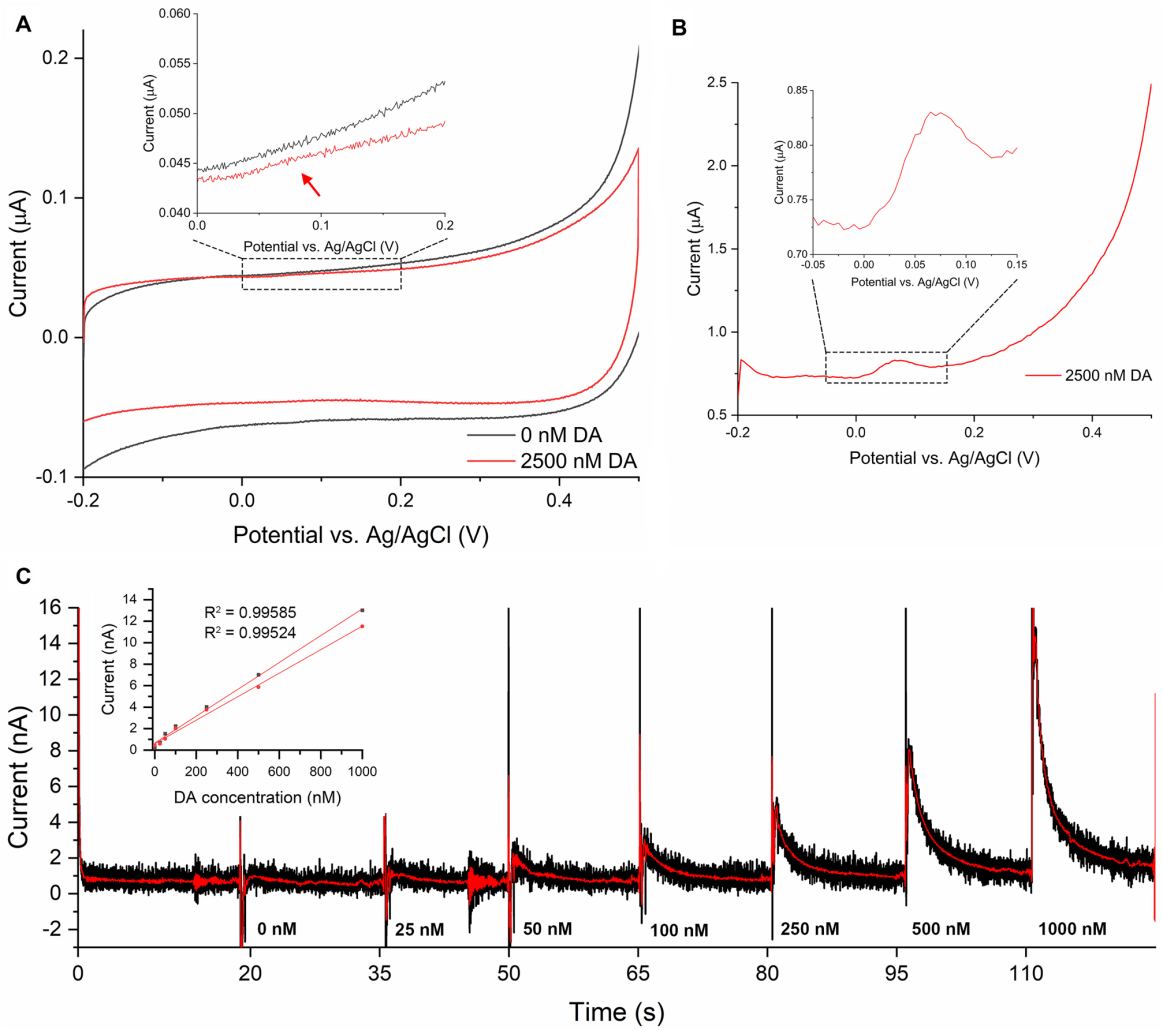
Dopamine at concentrations of 25–1000 nM can be detected from organoid medium that initially contained 200 μM AA, as is shown with SWCNT electrodes. While the oxidation peak was barely visible with CV at 2500 nM dopamine (DA) concentration (**A**), SWV evidently showed DA oxidation peak at 82 mV (vs. Ag/AgCl) potential (**B**). Fully linear current-concentration relationship ($\hbox {R}^{2}$ > 0.995) was obtained for DA in organoid medium (**C**), illustrating that DA can be chronoamperometrically detected with high sensitivity despite the initial presence of AA at high concentration. FFT-filtering was used (red trace in **C**) to filter out noise and thus make the trace more illustrative. Ringing noise that is most visible prior to 0 and 50 nM DA solution injections was caused when the pipette tip was brought into electrolyte.

After studying AA oxidation rate in culture medium, we wanted to experiment whether chronoamperometry can be used to detect dopamine in physiologically relevant concentration range (25–1000 nM) from complete human midbrain organoid medium that initially contained 200 μM AA (Fig. 2). We selected chronoamperometry because it is highly sensitive but non-selective electrochemical detection technique. After incubating the organoid medium for 18 hours inside incubator, we first analyzed the oxidation potential of dopamine in organoid medium. We were unable to clearly detect freshly added dopamine below 1000 nM concentration with cyclic voltammetry (CV) and thus selected 2500 nM concentration for the analysis (Fig. 2A). We also used a more sensitive detection technique, square wave voltammetry (SWV), to confirm the presence of dopamine and to determine its oxidation peak potential, being 82 mV in the organoid medium (Fig. 2B). Next, we switched the electrode into clean organoid medium that was incubated for 20.5 hours, and recorded dopamine injection series (25–1000 nM) with chronoamperometry (Fig. 2C). We performed this experiment twice and detected nearly fully linear ($\hbox {R}^{2}$ > 0.995) current-concentration relationship with the electrode both times.

Dopamine concentration in the CV and SWV measurements was larger than that found in physiological conditions, however it was purposefully selected because our aim was simply to find the dopamine oxidation potential in order to determine holding potential for chronoamperometry. In chronoamperometry, we injected 10 μL of organoid medium that contained only very minor amounts (25–1000 nM) of freshly added dopamine, causing the concentration of dopamine to decrease rapidly as it diffuses out from electrode surface. Although the slope of linear fit deviated slightly between two measurements (Fig. 2C inlet), we must emphasize here that injections of 10 μL volume were done manually and thus both the vicinity of pipette tip to electrode surface but also the injection pressure may deviate slightly between measurements. These deviations can cause minor differences in the recorded currents between individual experiments.

Although dopamine peak potential was at 82 mV, the onset to dopamine oxidation started at 50 mV, as was recorded with CV at 2500 nM concentration (Fig. 2A). For AA, the peak oxidation potential was at 394 mV but onset to AA oxidation started at around 50 mV in culture medium (Supplementary Figs. S4 and S5). Because of this, AA would generate oxidation current at the 125 mV (vs. Ag/AgCl) potential where we kept electrodes during chronoamperometric recording. Thus, AA would interfere dopamine detection because the electrodes were kept submerged in organoid medium during the dopamine injection series. If there was AA present at significant concentration in the medium, we would expect to observe a stable AA oxidation current that would be larger than that arising from dopamine oxidation. The current eventually decreased back to background level after dopamine injection, indicating that AA was no longer detectable with chronoamperometry.

N2B27 medium is the basic component of the organoid medium and is essentially identical to it but lacks AA, dbcAMP and growth factors in small concentration. Thus, comparison between N2B27 and organoid medium can be done. Because of the early onset of AA oxidation at 50 mV (vs. Ag/AgCl), it is apparent that separation as large as 300 mV in oxidation *peak* potentials between the analytes may not always be sufficiently large to provide selective analyte detection. After analyzing the CV curves of AA in N2B27 medium by selecting the potential-of-analysis to 125 mV (Supplementary Figs. S4–S6), we observed that AA oxidation current was 6.1 ± 2.0 nA after 6h, 0.9 ± 0.6 nA after 8 hours, and below 0 nA after 24 hours.

Thus, chronoamperometry with 125 mV holding potential can be used to measure dopamine from complete organoid medium after 8 hours, once the AA oxidation current has decreased to be negligibly small on our SWCNT electrodes. We must emphasize here that different electrode materials may show different onset and peak potentials for AA and dopamine, and that different culturing media likely also shift these potentials slightly. Our SWCNT electrodes were not selective, which is obvious from the fact that AA was oxidized at potentials below the dopamine peak oxidation. Despite the lack of selectivity and without making any surface modifications, we were able to record dopamine at physiologically relevant concentration from organoid medium due to the decay of AA.

Lastly, we must note that bicarbonate buffer containing cell culture media undergo alkalinity shift without the presence of 5% $\hbox {CO}_{2}$ in atmosphere^33^. Because dopamine oxidation mechanism is dependent on pH^34,35^, we performed all cell culture medium recordings within 30 minutes to avoid excess alkalinity shift from occurring. Accordingly, we did not observe a notable change in the color of organoid medium that contained phenol red as pH indicator (data not shown). We also tested the medium with pH paper and observed that the pH remained below pH 8 (data not shown).

## Conclusion

Here in this study, we showed that the interference coming from L-ascorbic acid in cell culture media can be completely eliminated by experimental design. After waiting for the decomposition of AA, neurotransmitters can be detected without the requirement of high selectivity between AA and analyte-of-interest. The results of this study provide means for designing experiments in a manner, where AA interference does not have to be considered. For example, long-term brain organoid studies may require daily recordings of dopamine to study developmental stage or effectiveness of given treatments. The researcher might purposefully select to capture data between 16-24 hours after medium change, allowing daily monitoring of the system also with electrodes that would not otherwise fully remove the interference from AA.

Lastly, we showed in complete human midbrain organoid medium that dopamine can be detected in physiologically relevant concentration range (25–1000 nM) with fully linear current-concentration relationship. While dopamine has earlier been detected in physiological concentrations from highly clean electrolytes such as PBS or aCSF, these clean electrolytes do not contain various molecules that adsorb to electrode surface and compete with dopamine for the electrochemically active surface sites, which would result into decreased sensitivity. Here in the present article, we show for the first time that dopamine can also be detected in physiologically relevant concentrations from a highly complex electrolyte such as cell culture medium. We must also emphasize that we selected a culture medium that can be directly used to culture human midbrain organoids or neurons, thus replicating most accurately the real in vitro conditions.

In order to study whether ascorbic acid is oxidized by the presence of oxygen alone, we compared the time-concentration profile of AA in PBS and cell culturing medium. We observed that electrochemical AA oxidation current remained highly stable in PBS over 8 hours, indicating very little of oxygen-induced AA oxidation. In contrast, most of the AA was already oxidized after few hours in culturing medium, which was observed by rapidly declining electrochemical oxidation current. By acknowledging the decay profile of AA, neurotransmitters can be detected with high sensitivity even if the culturing medium would be initially supplemented with AA. We showed this by measuring dopamine in the physiological concentration range (25–1000 nM) from complete culturing medium that was incubated for 20.5 hours and initially contained 200 μM AA.

## Materials and methods

### Preparation of electrodes

SWCNT networks were fabricated with earlier introduced protocol^28^. Briefly, the SWCNT networks were fabricated in a laminar flow reactor by chemical vapor deposition and were collected using a membrane filter.

Electrode sample manufacturing protocol for the SWCNT network electrodes is described as follows. The SWCNT network was press-transferred onto polymer substrate, after which the network was densified by adding 99.7% ethanol (Anora) and letting it evaporate in air. Electrical contact was made by adding conductive silver paste (Electrolube) and drying overnight in air, after which a conductive copper tape (Ted Pella) was used to contact the silver paste to copper slide. An inert PTFE-tape (Irpola) was used to insulate the electrode from other regions except a 2-mm hole that was earlier cut to the PTFE tape.

Tetrahedral amorphous carbon (ta-C) sample was prepared by protocol introduced earlier^26^. Briefly, 20 nm of titanium was sputtered onto p-type boron doped Si <100> wafer (R < 0.005 ohm-cm), after which 15 nm of (ta-C) was grown with filtered cathodic vacuum arc deposition. The wafer backside was scratched with diamond pen, after which it was scraped with copper piece to obtain a thin copper layer onto the surface. Next, conductive silver paste was added to the wafer backside and it was let dry in air for 15 minutes prior to being contacted with the copper slide. We also used conductive copper tape to provide alternative conductive path form the sample surface directly to copper slide. We insulated the electrode with PTFE-tape, similarly to the SWCNT electrodes.

Lastly, the copper slides of all electrode types were bent to fit the electrode into 12 well plate.

### Electrochemistry

All electrochemical procedures were carried out with Reference 600+ potentiostat (Gamry Instruments), using sterile 12-well plates (VWR) as the container for electrolytes that were maintained at 37 °C temperature throughout all procedures. A platinum wire was used as counter electrode (99.95% purity, Alfa Aesar), whereas we prepared an Ag/AgCl pseudoreference electrode by treating a silver wire (99.9% purity, Alfa Aesar) in 10% sodium hypochlorite solution (FF-Chemicals) for 45 minutes. We did not deareate the electrolyte solutions in order to recapitulate the real system where biological samples are measured.

All solutions were freshly prepared prior to being used, unless otherwise stated.

#### Electrolytes in ascorbic acid oxidation study

The electrolyte solution in our electrochemical experiments was either Dulbecco’s Phosphate Buffered Saline (PBS, Gibco), or N2B27 culture medium that can be used to culture human midbrain organoids^8^ when AA, growth factors and dibutyryl cyclic adenosine monophosphate (dbcAMP) are also added. The culture medium N2B27 was prepared according to earlierly introduced protocol^8^. A mixture having 1:1 ratio of DMEM/F-12 (Gibco) and Neurobasal (Gibco) media was prepared, which was supplemented by N-2 supplement (17502-048, Gibco) at 1:200 dilution, B-27 supplement (12587-010, Gibco) at 1:100 dilution, GlutaMAX supplement (Gibco) at 1:100 ratio, and Penicilin-Streptomycin (Gibco) at 1:100 dilution.

L-ascorbic acid (99.0–100.5% purity, Sigma) was dissolved into PBS to produce 20 mM AA stock solution, which was sterile-filtered once through 0.2 μm filter. The stock solution was stored at a -21 °C freezer in small aliquots to prevent multiple freezing-thawing cycles. Prior to creating electrolyte solutions with AA, the stock solution was thawed and it was added at 1:100 dilution into PBS and N2B27 medium to have 200 μM concentration in the solutions.

After preparing the electrolyte solutions and they were administered onto 21 $\hbox {cm}^{2}$ culture dishes (VWR) with or without AA, they were placed into a humidified incubator (37 °C, 5% $\hbox {CO}_{2}$, saturated humidity, RH 80-100%) until being measured. PBS solutions were measured at time points of 0h, 30 min, 1h, 2h, 4h, 6h, 8h after beginning of incubation, whereas N2B27 solutions were measured at same time points but also after 24h. The 0h time point measurement was done by taking an electrolyte-containing culture dish from the incubator, measuring the background without AA first, and then measuring the same solution after adding 20 mM L-ascorbic acid solution at a 1:100 dilution and thorough mixing. Electrolyte solution with freshly added AA at concentration of 200 μM was also measured after the measurement series in order to show that the decreasing oxidation current does not occur due to decreased electrode surface reactivity.

#### Electrolytes in dopamine detection

After studying AA decomposition profile, we prepared complete supplemented medium that can be used to culture human midbrain organoids, thus labelled as organoid medium. This organoid medium was prepared according to earlierly introduced protocol^8^. N2B27 was used as the base medium and it was prepared beforehand. Prior to being used as the organoid culturing medium, N2B27 medium was further supplemented with 10 ng/mL human glial cell line-derived neurotrophic factor (hGDNF, Peprotech), 10 ng/mL human brain derived neurotrophic factor (hBDNF, Peprotech), 1 ng/mL transforming growth factor beta 3 (TGF-$\beta$3, Peprotech), 500 μM dbcAMP (Sigma), and 200 μM ascorbic acid (Sigma). Supplements for the complete organoid medium were a kind gift by PhD Gemma Gomez-Giro from Prof. Jens Schwamborn’s laboratory.

This organoid medium was placed into 21 $\hbox {cm}^{2}$ culture dishes and was incubated overnight prior to being used in cyclic voltametry and chronoamperometric measurement for detecting dopamine. In cyclic voltammetry experiments, the medium had spent 18 hours inside incubator. Dopamine hydrochloride (Sigma) was then dissolved into PBS at a concentration of 99 μM, creating a fresh stock solution, after which this stock solution was diluted into organoid medium at concentrations of 500, 1000 and 2500 nM.

Chronoamperometric dopamine detection experiment contained injection-solutions of dopamine in organoid medium at various concentrations (0, 25, 50, 100, 250, 500, 1000 nM). In order to prevent alkalinity shift in the solutions, we prepared them immediately before performing the experiment. Organoid medium had spent 20 hours in incubator prior to administrating it into seven Eppendorf vials. A solution of 49.6 μM dopamine in PBS was added to each of the vials except the 0 nM dopamine vial, creating individual solutions with the desired diluted dopamine concentration.

#### Electrochemical treatment

Prior to using the SWCNT sheet electrodes, their surfaces were treated by electrochemical oxidation at 1.5 V (vs. Ag/AgCl) potential for 30 seconds in PBS, followed by a second treatment at 0.4 V (vs. Ag/AgCl) for 60 seconds to stabilize the surface. The ta-C electrodes did not undergo any electrochemical treatments, and were used as such.

#### Cyclic voltammetry

Cyclic voltammetry was used to measure the concentration of AA at given time points (0 h, 30 min, 1 h, 2 h, 4 h, 6 h, 8 h, 24 h), having either PBS or N2B27 medium as the electrolyte in AA oxidation experiments. In the dopamine detection experiment where cyclic voltammetry was used to find dopamine oxidation potential, organoid medium was used as the electrolyte. Scan rate was 400 mV/s in both experiment types.

#### Square wave voltammetry

Square wave voltammetry (SWV) was used between -0.2 and 0.5 V (vs. Ag/AgCl) to more accurately determine the oxidation potential of dopamine in organoid medium. Because the shape of SWV voltammograms may be significantly altered by experimental parameters, they are listed here as follows: Pulse Size 50 mV, Frequency 50 Hz, Step Size 5 mV, Max Current 0.006 mA.

#### Chronoamperometry

Chronoamperometry was used for the detection of dopamine in physiologically relevants concentrations (25–1000 nM) in organoid medium. We selected holding potential of 125 mV (vs. Ag/AgCl) based on the data obtained in SWV for dopamine oxidation.

Injection solutions were prepared as described earlier. Prior to injecting any solutions to the electrode surface, we waited for 20 seconds until the electrode had reached equilibrium state with the electrolyte. We selected 10 uL as the injection volume and administered this amount by manual pipetting onto the electrode that was maintained submerged in organoid medium. The injection series was performed from smaller dopamine concentrations to larger concentrations (0 nM $\rightarrow$ 1000 nM), and we waited for 15 seconds between each injection to let the previous injection solution diffuse away from electrode surface.

#### Data processing

Measurement data was analyzed in Echem Analyst software (Gamry). We observed that background current from the electrolyte itself distorts fitting (data not shown) to pseudo first-order chemical reaction equations (Supplementary Eqs. S3–S6). Thus, all the data related to AA oxidation was background-subtracted, meaning that the oxidation current arising from the electrolyte itself (PBS or N2B27, without AA) was subtracted away. In mathematical terms, this would be described as ${\text{I}}_{{{\text{AA-Oxidation}}}} = {\text{I}}_{{{\text{AA-Measured}}}} - {\text{I}}_{{{\text{Electrolyte}}}}$. Because this background current is dependent on the potential where it is analyzed from, we selected to analyze background oxidation current from the same potential where AA oxidation current was analyzed from.

After obtaining the oxidation currents for AA and dopamine, the data values were transferred to Origin software (version 2021b, OriginLab). Descriptive statistics tool was used to obtain mean average and standard deviation (SD) values from the data sets. We plotted the mean values with SD into graphs, after which fitting was done with either linearized fit model or pseudo first-order chemical reaction equation. Fit equations are shown in the graphs for pseudo first-order chemical reaction, whereas linearized fit equation (y = a + b*x) is not shown.

Chronoamperometric trace shown in Fig. 2C also contains a smoothed trace obtained by performing Fast Fourier Transformation (FFT) smoothing to the raw data. Smoothing was done based on low-pass parabolic filter with Cutoff frequency at 10 Hz and selecting Points of Window to value 5.

## Supplementary Information

Supplementary Information.

## Data Availability

The raw data required to reproduce these findings alongside with our data processing file are available to download from [Rantataro, Samuel (2022), “Ascorbic acid does not necessarily interfere with the electrochemical detection of dopamine”, Mendeley Data, V1, Doi: 10.17632/3yffvswj4r.1].
