# Supplementary material for: Ascorbic acid does not necessarily interfere with the electrochemical detection of dopamine
Source: Sci Rep. 2022 Nov 23;12:20225. doi: 10.1038/s41598-022-24580-0 (PMC9684410; doi:10.1038/s41598-022-24580-0)
Supplement: Supplementary file 1 — Supplementary Information. [file 41598_2022_24580_MOESM1_ESM.pdf]

Ascorbic acid does not necessarily interfere with the electrochemical detection of dopamine - Supplementary Information

Samuel Rantataro[1,*], Laura Ferrer Pascual[1], and Tomi Laurila[1,2]

[1]Aalto University, Department of Electrical Engineering and Automation, Espoo, 02150, Finland
[2]Aalto University, Department of Chemistry and Materials Science, Espoo, 02150, Finland
[*]samuel.rantataro@aalto.fi

ABSTRACT

This supplementary information contains equations and additional figures that supplement the main manuscript.

Equations for electrochemical reactions

Although AA oxidation is a multi-step process, it eventually oxidizes irreversibly to dehydroascorbic acid (DHAA)[1] and we may model the oxidation of AA as a common irreversible chemical reaction:

$$A + B \rightarrow oxidation\ products \tag{1}$$

, where reagent A denotes ascorbic acid and reagent B denotes the sum of all the oxidative reagents.

As reasoned and stated in the main article, we assume that the oxidation reaction of AA, induced by the culture medium, can be modeled with pseudo first-order chemical reaction equation[2] (Equation 2):

$$-\frac{d[A]}{dt} = k_{exp}[A] \tag{2}$$

, where [A] denotes the concentration of AA and k_{exp} denotes experimentally determined rate constant.

Because this equation has the same form as first-order reaction, we may modify its structure similarly to first-order reaction[2]. After integration over the limits [A] = $[A]_0$ and time point t = 0 to t, we can obtain:

$$ln[A]_t - ln[A]_0 = -k_{exp}t \implies e^{ln[A]_t} = e^{ln[A]_0 - k_{exp}t} \implies [A]_t = [A]_0 e^{-k_{exp}t} \tag{3}$$

, where $[A]_t$ denotes concentration at time point t, $[A]_0$ concentration at time point zero, and k_{exp} denotes the pseudo first-order reaction rate. We must note here that although AA oxidation is a series of several electron and proton transfer steps[1], we may study the system with pseudo first-order reaction kinetics if one of the reactions is much slower than others: the slowest reaction will be the rate-determining step[3]. We also assume that AA oxidation reaction only requires one AA molecule and thus the electrochemical reaction order (ν) with respect to AA concentration is equal to 1, indicating that there is a one-to-one correspondence[4] between occurrence of the rate-determining reaction and overall oxidation reaction of AA to DHAA.

Thus, oxidation current is directly proportional to concentration of the analyte at electrode surface[5]:

$$I_{Oxidation} = nFAk_b * C_{Analyte} \implies C_{Analyte} = \frac{I_{Oxidation}}{nFAk_b} \tag{4}$$

, where n is the number of transferred electrons in electron transfer reaction, F is the Faraday constant, A is the electrode area, k_b is the reaction rate of oxidation, and $C_{Analyte}$ is the concentration of reduced form of analyte at electrode surface.

Because concentration $C_{Analyte}$ in the Equation 4 is essentially same as [A] in Equation 3, and electrochemical order of the reaction with respect to analyte concentration (here AA) is one as indicated above, we can relate current of the oxidation reaction to the AA concentration as follows:

$$C_{Analyte} = \frac{I_{Oxidation}}{nFAk_b} = [A] \tag{5}$$

By combining Equations 3 and 5, we can obtain the relationship between oxidation current and pseudo first-order reaction rate k, as follows:

$$\frac{I_t}{nFAk_b} = \frac{I_0}{nFAk_b} * e^{-k_{exp}t} \xrightarrow{\text{*nFAk}_\text{b}} I_t = I_0 * e^{-k_{exp}t} \tag{6}$$

, where I_t denotes oxidation current at time point t, I_0 denotes oxidation current at time point zero, and k_{exp} denotes the pseudo first-order reaction rate, respectively.

Thus, we can analyze the pseudo first-order reaction rate for AA oxidation in culture medium based on AA oxidation current at given time points.

References

1. Du, J., Cullen, J. J. & Buettner, G. R. Ascorbic acid: Chemistry, biology and the treatment of cancer. *Biochimica et Biophys. Acta - Rev. on Cancer* **1826**, 443–457, DOI: 10.1016/j.bbcan.2012.06.003 (2012).

2. Jordan, R. B. *Reaction Mechanisms of Inorganic and Organometallic Systems* (Oxford University Press, 2007), 3rd edn.

3. Engel, T. *Physical Chemistry* (Pearson Education limited, 2013), 3rd edn.

4. Bockris, J. O., Reddy, A. K. N. & Gamboa-Aldeco, M. *Modern Electrochemistry 2A* (Springer, 2000), 2nd edn.

5. Bard, A. J. & Faulkner, L. R. *Electrochemical Methods: Fundamentals and Applications* (John Wiley and Sons Inc., 2001), 2nd edn.

Supplementary figures

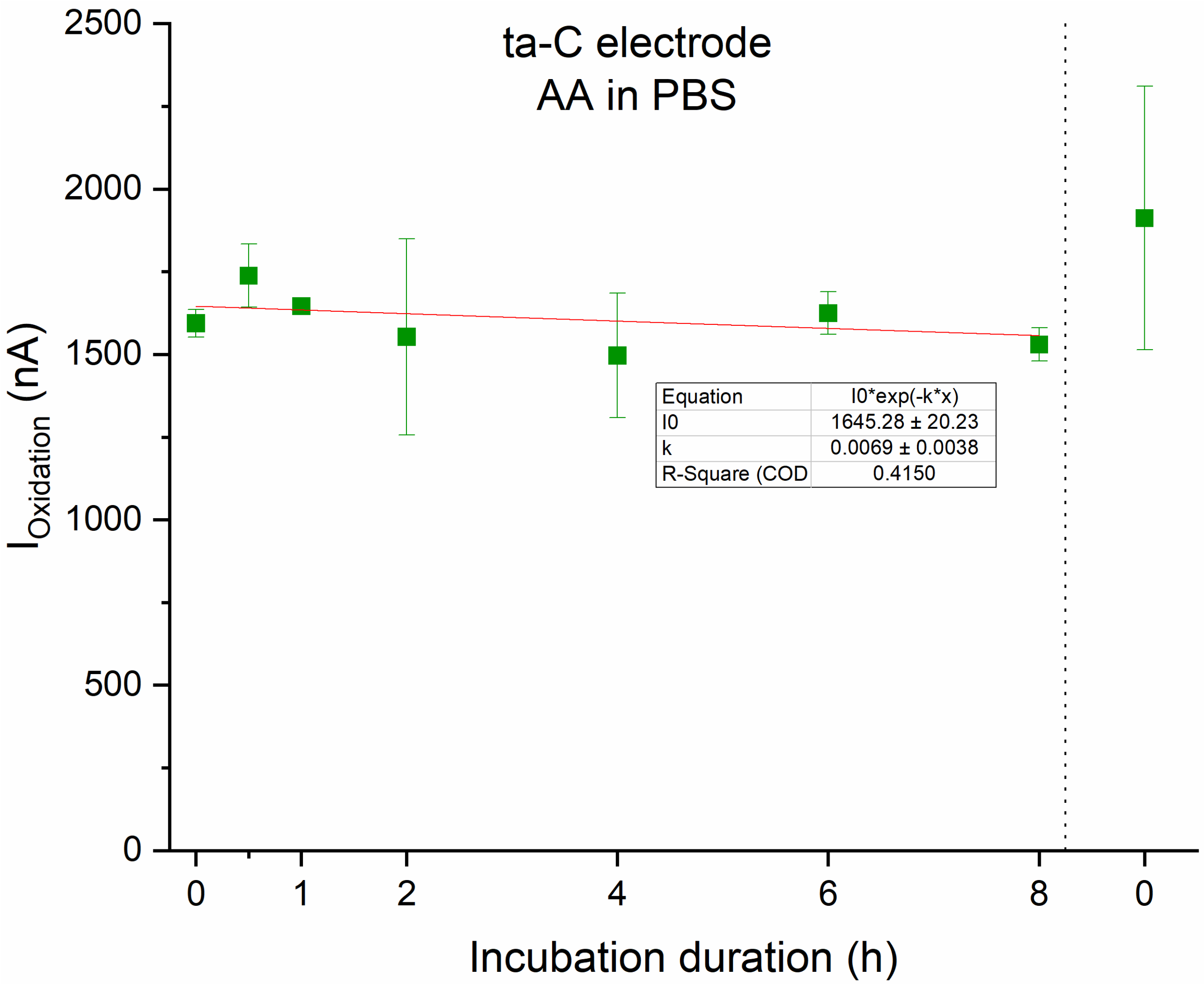

Figure 1. The concentration of ascorbic acid remains stable also on ta-C electrodes. The relationship between AA concentration and incubation did not follow the first-order chemical reaction rate, as is illustrated by poor fit ($R^2 = 0.415$) to the equation. Significantly increased current at the second t = 0 measurement was surprising, which was caused by notably increased current in one of the electrodes. This increase was likely due to increased reactive surface area, which may occur if the PTFE-tape peels slightly off the electrode surface due to loss of adhesion around the 2 mm hole. Thus, the error was arising from sample manufacturing but does not affect the analysis because AA oxidation current was almost the same at 8 h and 0 h time points.

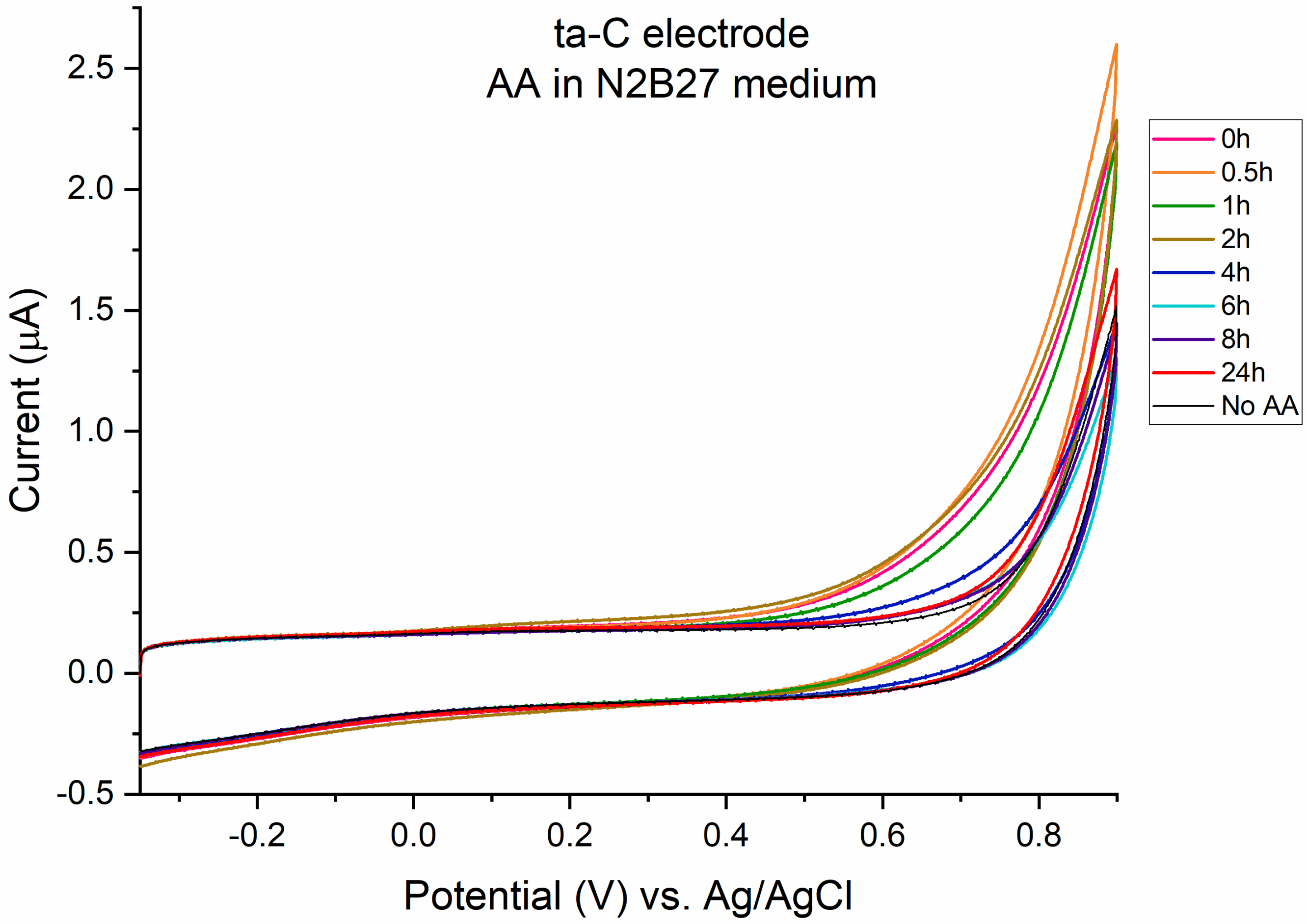

Figure 2. ta-C electrode shows significant oxidation of medium components at potentials below ascorbic acid oxidation, making accurate determination of AA oxidation current impossible. While one can see decreasing oxidation current, for example when analyzed at potential of 0.7 V (vs. Ag/AgCl), there is notable deviation from linearity due to following inconsistencies: $I_{Oxidation}$, t = 0.5h > $I_{Oxidation}$, t = 0h ; $I_{Oxidation}$, t = 2h > $I_{Oxidation}$, t = 1h ; and $I_{Oxidation}$, t = 8h > $I_{Oxidation}$, t = 6h . Because of this, we did not analyze AA oxidation in N2B27 medium with ta-C electrodes.

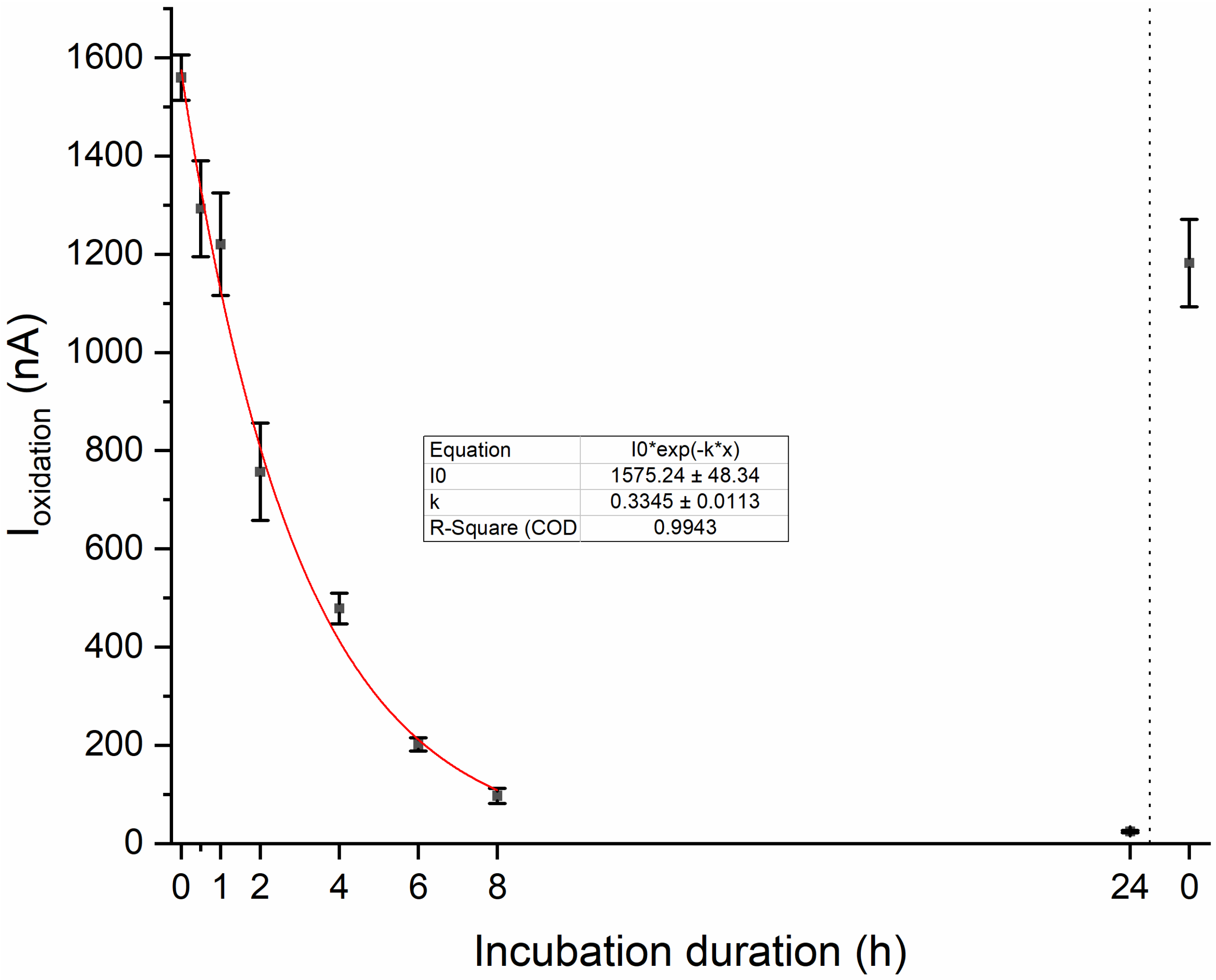

Figure 3. Concentration of AA decreases in N2B27 medium as a function of first-order chemical reaction, as is illustrated with near-perfect fit ($R^2 > 0.994$) between incubation time 0-8 hours. Oxidation current from freshly added AA was measured again after the measurement series to show that the decrease in oxidation current is not arising simply from fouling of the electrode surface. This second data point with "0h incubation" is shown on the right side of dotted line. Oxidation current was analyzed from AA oxidation peak potential. Electrode type was SWCNT.

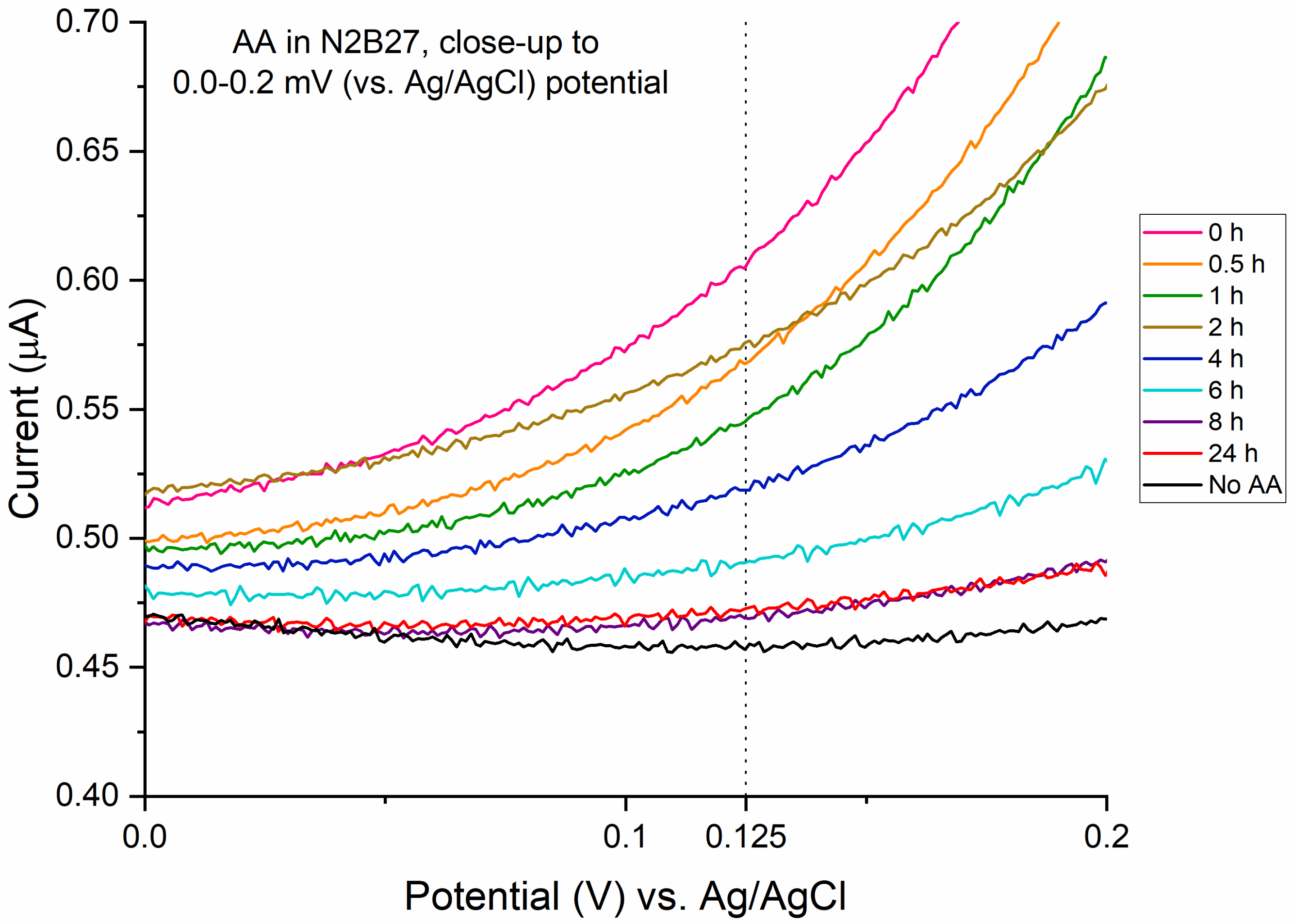

Figure 4. Cyclic voltammogram of 200 μM AA in N2B27 medium, close-up to potential range 0-200 mV (vs. Ag/AgCl). Dashed vertical line represents the holding potential that was used in chronoamperometric detection of dopamine. Significant AA oxidation current can be detected at time points below 6 hours. Electrode type was SWCNT.

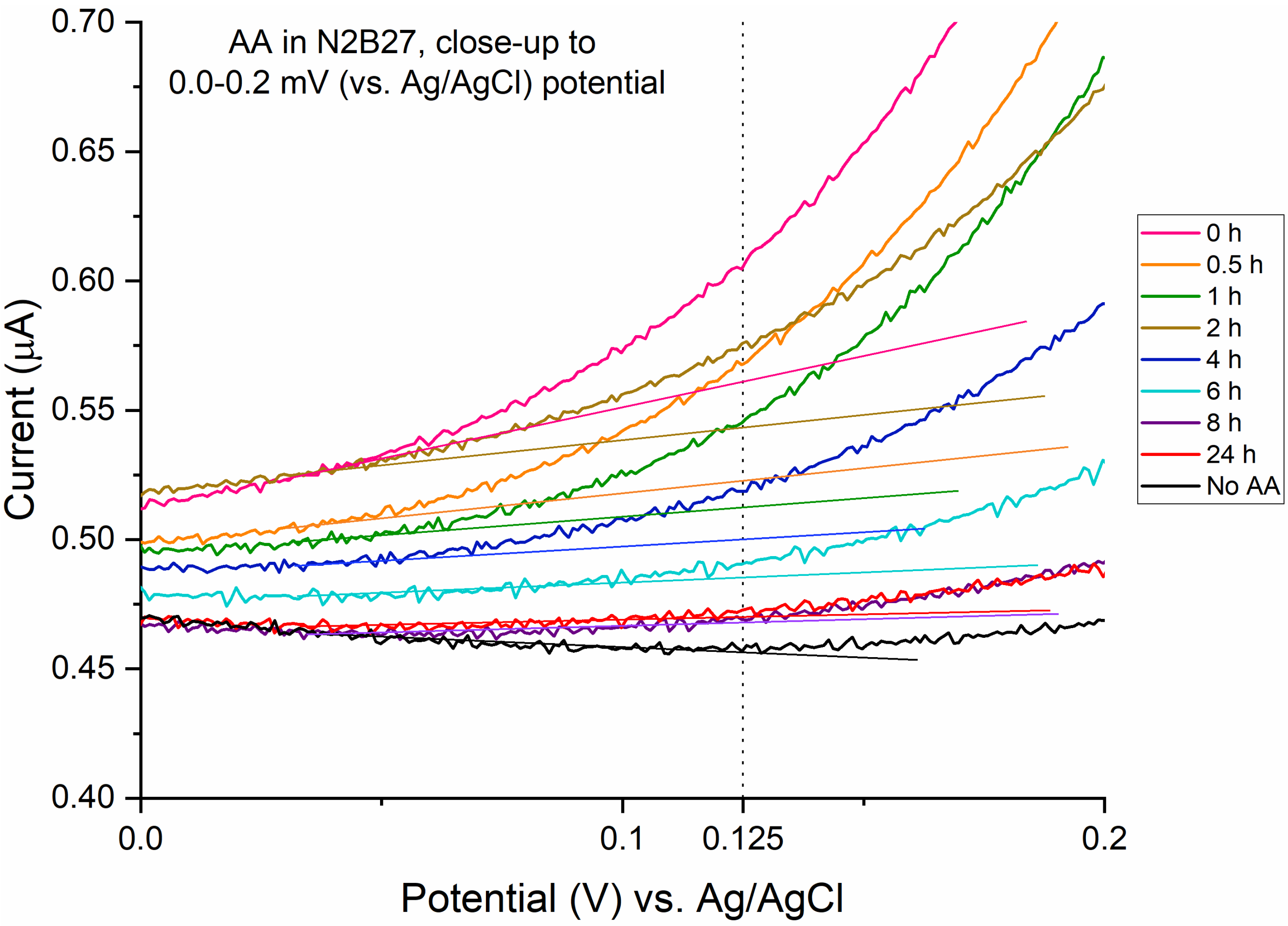

Figure 5. Cyclic voltammogram of 200 μM AA in N2B27 medium, with drawn baselines according to which the AA oxidation current can be analyzed from. Dashed vertical line represents the holding potential that was used in chronoamperometric detection of dopamine. At 125 mV (vs. Ag/AgCl) potential, the AA oxidation current is almost zero with incubation durations larger than 8 hours. Electrode type was SWCNT.

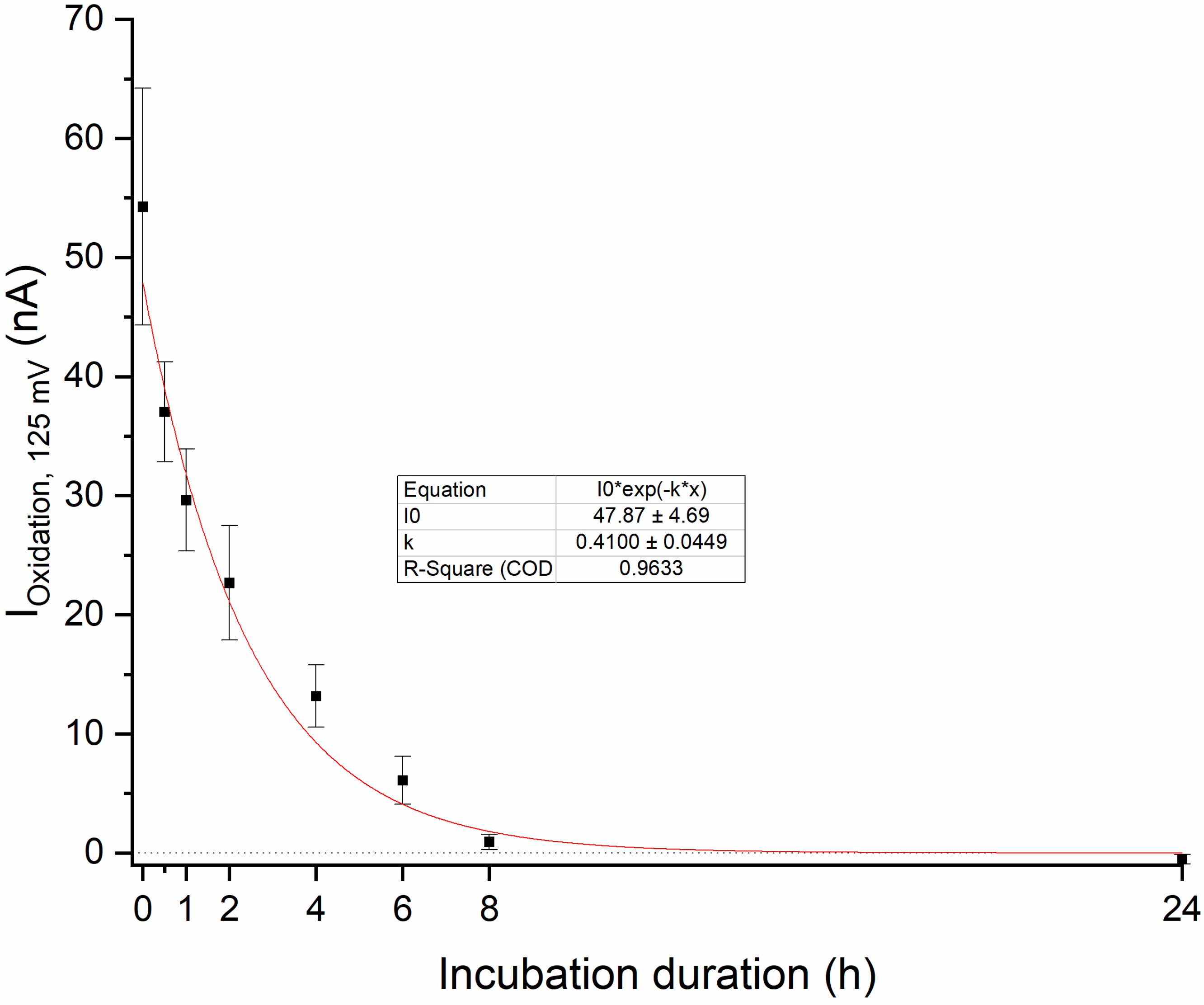

Figure 6. Oxidation current of AA in N2B27 medium is notably smaller when analyzed from 125 mV (vs. Ag/AgCl) potential instead of AA oxidation peak potential. After incubation of 8 hours, the current is only 0.933 nA. The concentration of AA remains to follow the function of first-order chemical reaction, however with smaller goodness of fit ($R^2 = 0.9633$). This decrease in goodness of fit occurs because the oxidation current is smaller, thus causing proportionally increased error from baseline drawing. Currents are background subtracted, meaning the oxidation current arising from the medium without AA is subtracted out. Electrode type was SWCNT.
